# Tantalum Dental Implants: A New Frontier in Biocompatibility and Bone Integration

**DOI:** 10.7759/cureus.101497

**Published:** 2026-01-13

**Authors:** Ankita Sharma, Vikas Kumar, Gyan Vardhan

**Affiliations:** 1 Prosthodontics and Implantology, Adesh Institute of Dental Sciences and Research, Bathinda, IND; 2 Pharmacology, All India Institute of Medical Sciences, Bathinda, Bathinda, IND; 3 Pharmacology, All India Institute of Medical Sciences, Rishikesh, Rishikesh, IND

**Keywords:** crown and bridge prosthesis, healthcare biomaterial, implant biomaterials, osseoincorporation, tantalum implant

## Abstract

Tantalum dental implants have emerged as a promising alternative to conventional titanium implants, offering superior biocompatibility, osteoconductivity, and enhanced osseointegration. The distinctive properties of tantalum, particularly its highly porous trabecular structure, closely mimic the natural architecture of bone and facilitate optimal bone ingrowth and vascularization. This porosity creates an ideal environment for osteocyte infiltration and cellular attachment, promoting robust bone-implant integration, which is essential for long-term implant stability and success.

Despite these advantageous biological properties, several significant challenges limit the widespread clinical adoption of tantalum dental implants. The high cost of raw tantalum material, and the requirement for specialized fabrication techniques - such as chemical vapor deposition and powder metallurgy - pose substantial economic and technical barriers.

Additionally, the paucity of long-term clinical data, concerns regarding potential material degradation in the oral environment, and complexities in implant design optimization remain unresolved. While preliminary clinical studies demonstrate encouraging outcomes, comprehensive research is imperative to address these limitations and establish evidence-based protocols.

This review critically examines the current state of tantalum implants in dental practice and provides insights into their potential role in advancing modern implantology.

## Introduction and background

Dental implants have revolutionized oral rehabilitation, providing an effective solution for patients with partial or complete tooth loss. Despite the high success rates reported for implant-supported prostheses, a small percentage (5%-10%) still fail [1,2]. Advances in implant surface modifications have been shown to enhance bone integration [3-5], and ongoing research continues to focus on improving the success and longevity of dental implants. Porous tantalum trabecular metal (PTTM) is increasingly utilized in dental implants due to its ability to enhance surface roughness and facilitate osseointegration - the direct growth of bone into the implant [6]. With a porosity of 80%, PTTM closely mimics the bone microstructure and exhibits similar elasticity [7,8]. The trabecular design of the implant increases surface area, promoting osseoincorporation through both bone ongrowth and ingrowth [9]. Research indicates that, while titanium fosters cell proliferation, tantalum plays a key role in osteoblastic differentiation [10].

**Figure 1:**
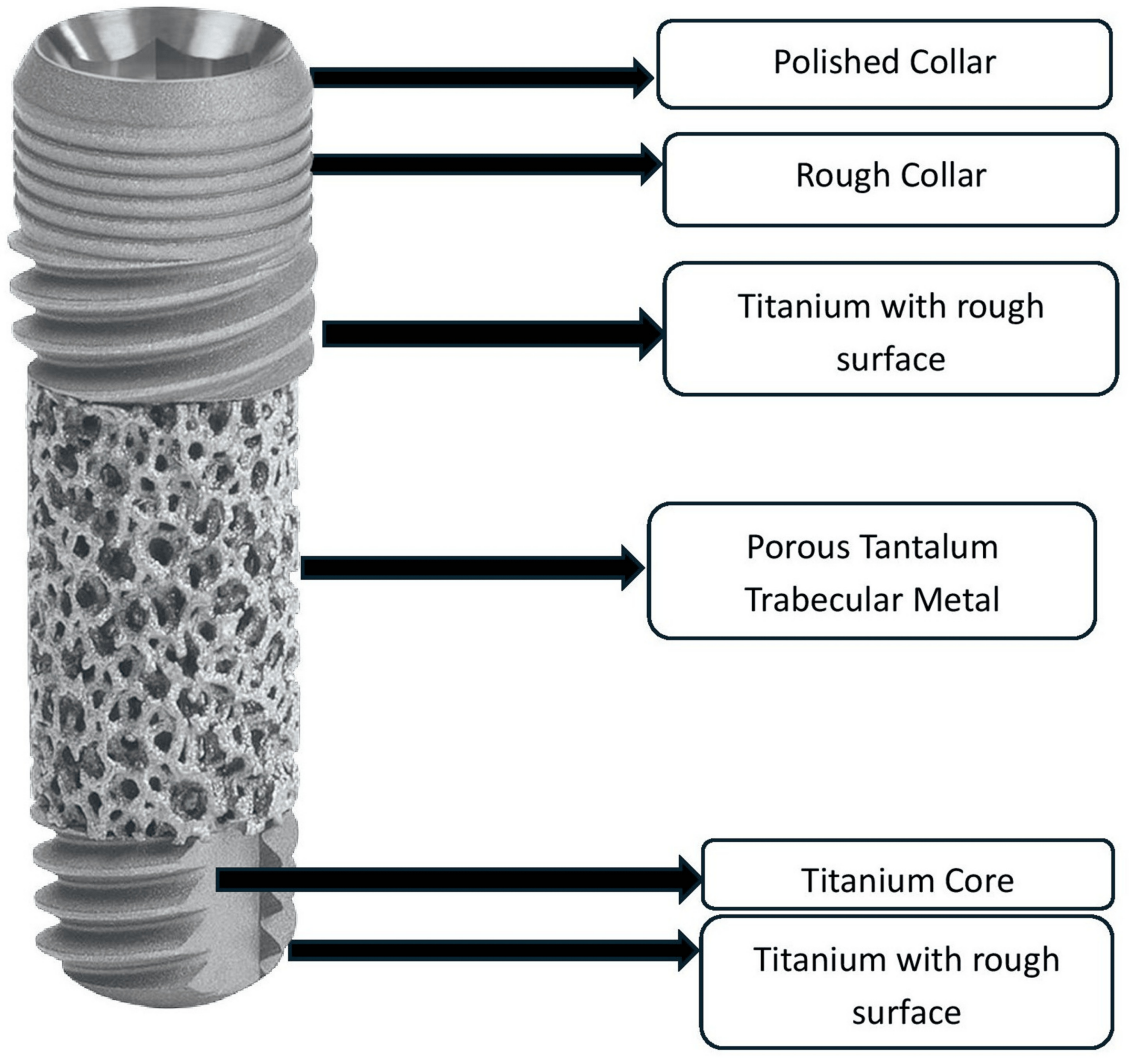
Schematic Structure of Tantalum Implant Credit: Image provided by ZimVie India

The porous structure of tantalum, resembling spongy bone, is believed to enhance bone ingrowth. PTTM's ability to stimulate neovascularization, wound healing, and osteogenesis has also been demonstrated in orthopedic implants. Thus, incorporating PTTM into dental implants offers significant potential for improving bone integration and ensuring the long-term success of the implant. The trabecular metal dental implant combines tantalum and titanium alloy to optimize functionality and osseointegration. Its midsection features a porous tantalum meshwork that supports neovascularization and bone formation directly into the implant, while the coronal and apical portions are screw-shaped and made from titanium alloy (Figure 1).

These titanium alloy surfaces undergo microtexturing with hydroxyapatite to further enhance osseointegration. The trabecular metal implant, resembling trabecular bone in structure, provides improved biomechanical properties and long-term stability. Although existing research has provided insights into healing patterns, integration, and bone response with the trabecular metal dental implant, our understanding of these processes in the PTTM-enhanced areas of the implant remains limited. For years, it has been documented that implant survival rates improve with osseointegrated implants. However, with modern advancements in osseoincorporation within implants, the longevity and stability of implants have increased. 

Tantalum possesses key characteristics, including a porous structure, strength, and durability, while having a favorable safety profile for clinical application. These qualities enable implants to facilitate crucial blood vessel development, which promotes the creation, growth, and preservation of new bone tissue. The metal's structural resemblance to spongy bone helps minimize stress-shielding effects and contributes to bone preservation. New bone development is vital for achieving the implant's biomechanical stability [10]. However, tantalum has drawbacks, including a high elastic modulus and expensive production costs. Consequently, it isn't typically the primary material choice for implant manufacturing, leading researchers to explore alternatives, such as porous tantalum or various alloy combinations, to enhance its physical characteristics. A comprehensive cost-benefit evaluation is necessary when deciding whether to proceed with tantalum-based solutions [11].

This literature review aimed to examine the utilization and application of tantalum in dental implantology, with a particular focus on its integration with bone tissue.

## Review

Literature review process

A search strategy was developed to conduct the literature review. The primary databases searched included PubMed/Medline, Scopus, Web of Science, Embase, and the Cochrane Library, with Google Scholar used as an additional resource for broader coverage. Keywords used in the search included “implant biomaterials,” “tantalum implant,” “osseoincorporation,” “prosthetic applications,” and “healthcare biomaterial.”

To ensure the relevance of the studies, filters were applied to include only peer-reviewed articles in English. The types of studies included clinical trials, systematic reviews, and case studies.

History of tantalum

Tantalum (Ta) is a rare transition metal with atomic number 73, characterized by exceptional resistance to corrosion. The term “tantalum” is derived from Tantalus, a figure from Greek mythology, condemned to eternal punishment, wherein water and fruit perpetually receded just beyond his reach [12]. This mythological reference reflects the “tantalizing” chemical behavior observed by early chemists: tantalum demonstrated remarkable inertness when exposed to most acids, reacting only with hydrofluoric acid and acids containing fluoride ions or sulfur trioxide [13].

Tantalum has been extensively utilized as an alloying element due to its high melting point and resistance to chemical exposure. The element was first identified in 1802 by the Swedish chemist Anders Gustav Ekeberg [14]. In the early period of its discovery, it was commonly encountered in its oxide form, referred to as columbium, occurring within the mineral assemblages columbite and tantalite [15]. Subsequently, the English chemist William Hyde Wollaston established that both columbite and tantalite were mineral sources of the same elemental species, leading to the retention of the name tantalum [16].

Porous tantalum trabecular metal (PTTM)

Despite tantalum’s excellent biocompatibility, chemical inertness, and corrosion resistance, its early application in implantology was constrained by difficulties in processing solid tantalum. Traditional bone replacement strategies have relied on solid metals, such as titanium, or porous ceramics, including hydroxyapatite and tricalcium phosphate, often as surface coatings on metal alloys. These approaches failed to replicate cancellous bone architecture and were associated with mechanical shortcomings, particularly inadequate yield strength and ductility of the coatings. The introduction of PTTM in the early 1990s addressed these limitations [17].

**Figure 2:**
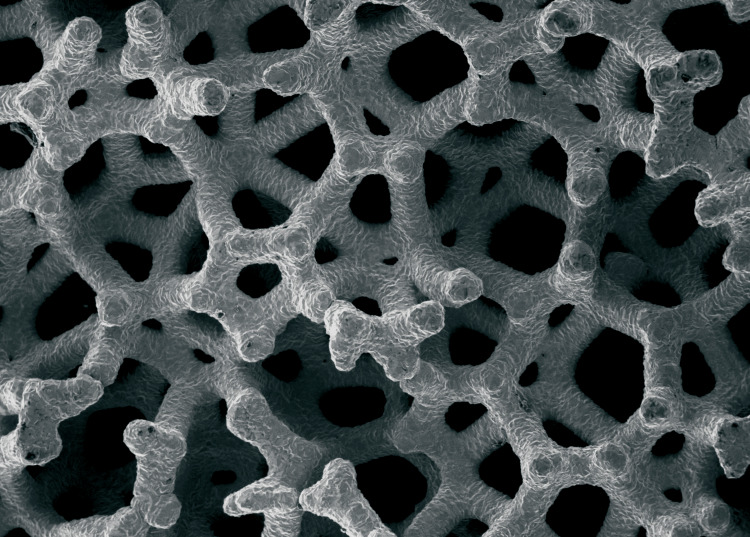
Dodecahedral Framework of Tantalum Credit: Image provided by ZimVie India

**Figure 3:**
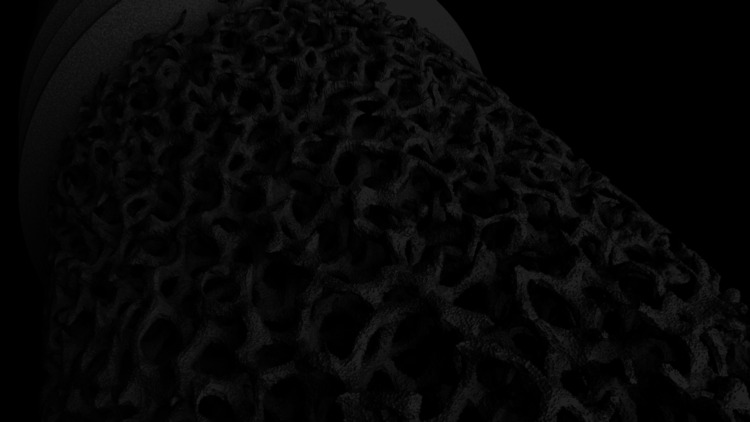
Three-Dimensional Dodecahedral Repeats of Tantalum Credit: Image provided by ZimVie India

PTTM is a porous biomaterial with an open-cell structure resembling trabecular bone, featuring three-dimensional dodecahedral repeats (Figures 2-3) [18]. These dodecahedral units are created using a foam-like vitreous carbon scaffold, which forms the initial framework and later becomes the internal skeleton of the PTTM implant. The vitreous carbon scaffold is placed in an air-sealed chamber. Unlike natural tantalum extraction, recycled tantalum - often used in industries like electronics - is employed in the PTTM manufacturing process. The tantalum coating is applied via a chemical vapor diffusion process involving hydrogen and chlorine gases, in which tantalum is evaporated as TaCl_2_ and deposited onto the scaffold. PTTM's high porosity makes it superior to other metal implants, such as titanium. The vitreous carbon skeleton, integral to the trabecular framework, can be modified to create various PTTM designs, which are particularly beneficial for orthopedic implants.

Design and manufacturing of tantalum implants

The three-dimensional porous tantalum enhancement on titanium dental implants facilitates both bone ongrowth and bone ingrowth, or osseoincorporation.

**Figure 4:**
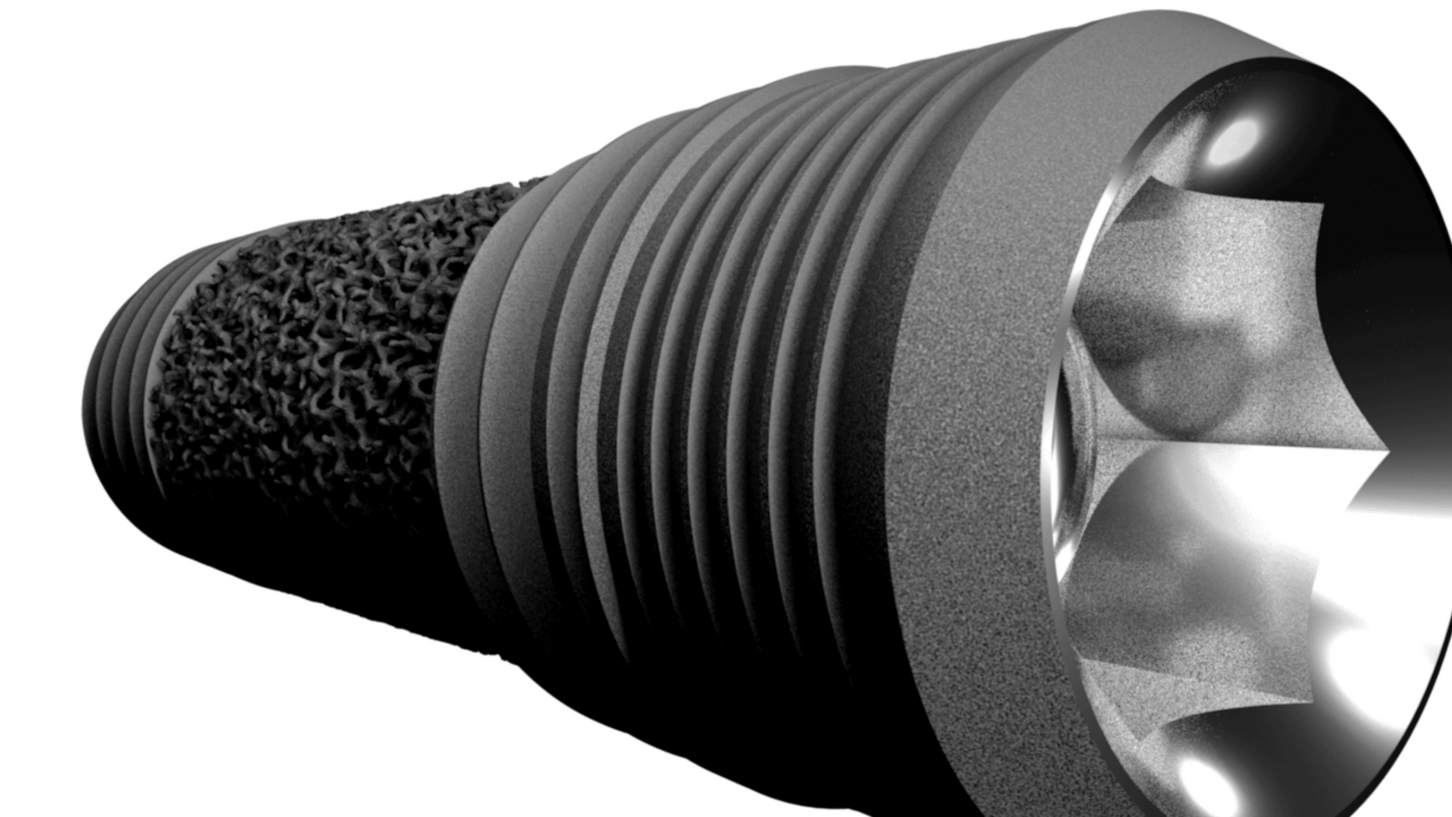
Three-Dimensional Porous Tantalum Trabecular Metal Design Credit: Image provided by ZimVie India

The implant, designed from titanium-tantalum alloys produced via additive manufacturing, offers distinct mechanical and biological benefits for implant applications; its integration with engineered lattice structures has not yet been fully explored [19,20]. Different lattices, such as three-dimensional lattice structures for biomedical implants using promising minimal surfaces - gyroid, diamond, and Schwarz primitive geometries - are employed (Figure 4). These lattices are fabricated through laser powder-bed fusion, using a blend of elemental titanium-tantalum powder [21].

Mechanism of integration of tantalum implants

Concept of Osseointegration

Osseointegration encompasses both the biological process and the resulting interface formed between the exogenous implant material and bone, wherein newly formed bone establishes direct contact with the implant surface. This bone-implant interface is critical for long-term implant stability and load transfer and underpins the clinical success of tantalum-based implant systems [22].

Concept of Osseoincorporation

PTTM exhibits excellent biocompatibility, osteoconductivity, and bone ingrowth, with positive outcomes in both in vitro and in vivo studies as well as human trials. Additionally, PTTM promotes bone ongrowth and ingrowth, facilitating neovascularization and new bone formation directly into the implant - a process referred to as "osseoincorporation" [9].

**Figure 5:**
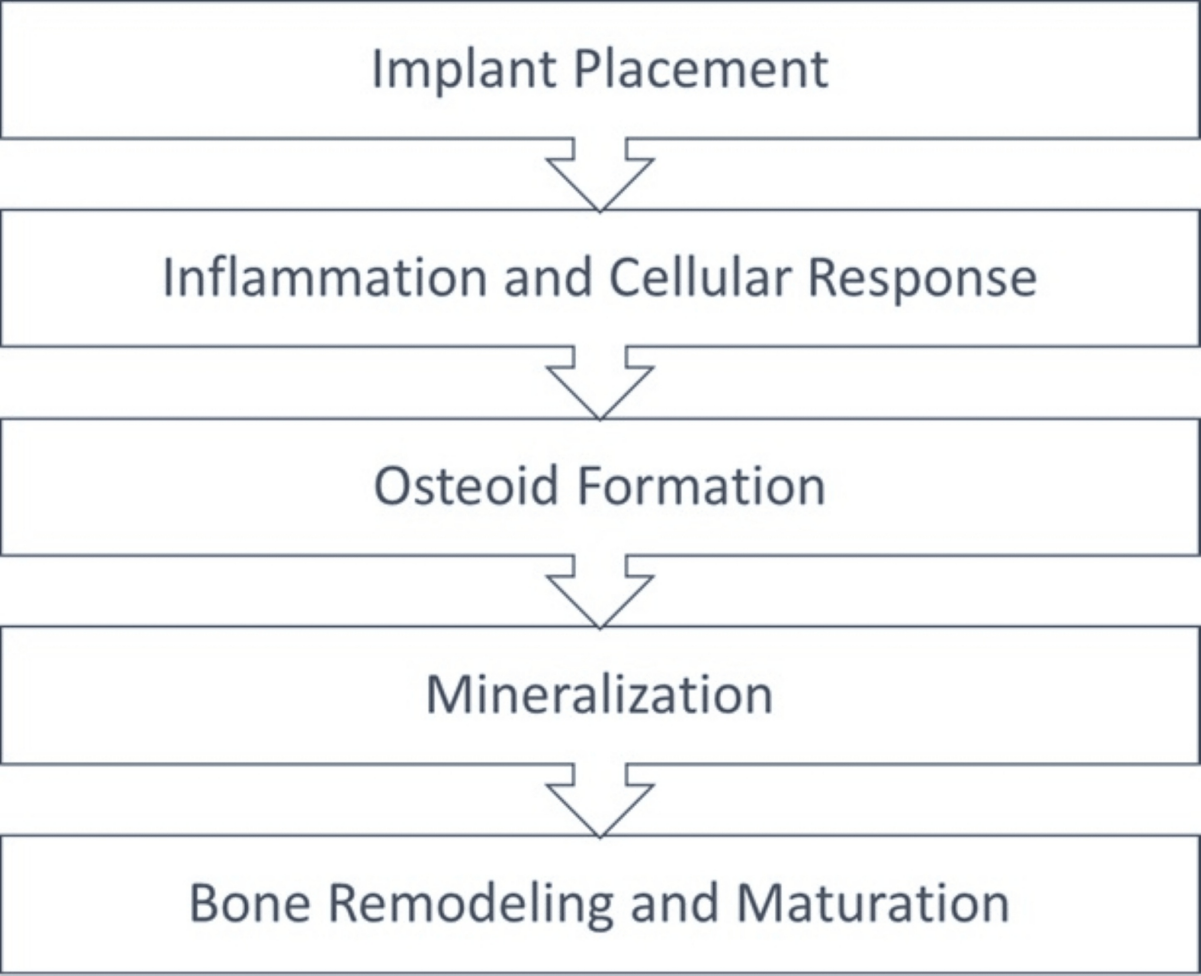
Stages of Osseoincorporation Credit: [9]

Mechanism of osseoincorporation: The stages of osseoincorporation are as follows (Figure 5): (i) Implant placement - When the implant is first placed into the bone, a blood clot forms around the surface of the implant. This blood clot contains growth factors, cytokines, and other molecules that help mediate the healing response. (ii) Inflammation and cellular response - The initial inflammatory phase begins, attracting immune cells to the site of implantation. These cells help clear any debris and initiate tissue repair. The inflammatory response also promotes the recruitment of mesenchymal stem cells and osteoblast precursors. (iii) Osteoid formation - As healing progresses, osteoblasts (bone-forming cells) migrate to the surface of the implant and begin producing an osteoid matrix, a soft, unmineralized form of bone. This matrix serves as a scaffold for the eventual mineralization and hardening of bone tissue around and within the porous implant surface. (iv) Mineralization - Osteoblasts continue to mineralize the osteoid matrix, leading to the formation of bone tissue around the implant. This mineralization process is essential for creating a robust bond between the implant and the surrounding bone. (v) Bone remodeling and maturation - Over time, the new bone tissue undergoes remodeling, in which both osteoblasts and osteoclasts (bone-resorbing cells) work together to shape and refine the bone structure around the implant. The implant surface may also undergo changes, such as the formation of a thin layer of bone around, as well as within, it, further enhancing the stability of the implant.

Histological Aspects of Tantalum Implants

Histological analysis reveals an area of enhanced bone formation within the tantalum mesh, with a notable increase in osteocyte infiltration. Vascular canal formation and partial bone trabeculae remodeling are also observed. The trabeculae are densely populated with osteocyte lacunae, which are predominantly spherical in shape and irregularly arranged, characteristic of woven bone [23,24].

Mechanistic basis for PTTM implants' success over traditional implants

PTTM implants exhibit enhanced osseointegration through complementary biological and biomechanical mechanisms. From a biological perspective, the three-dimensional trabecular architecture, characterized by a high porosity of approximately 75%-80%, promotes superior osseoincorporation by facilitating extensive bone ingrowth and vascular infiltration, when compared with conventional titanium implant surfaces. This interconnected porous network provides a favorable microenvironment for osteogenesis and sustained implant-bone integration.

From a biomechanical standpoint, tantalum demonstrates an elastic modulus of approximately 3 GPa, closely resembling that of cancellous bone and markedly lower than that of titanium (approximately 110 GPa) [25]. This modulus compatibility reduces stress shielding, enhances physiological load transfer, and supports more favorable peri-implant bone remodeling.

Collectively, the synergistic effects of osteoconductive scaffold architecture and biomimetic mechanical behavior underpin the accelerated osseointegration and long-term stability observed with PTTM implants. These mechanisms can be broadly conceptualized under two principal domains: biological adaptation and biomechanical interplay.

Biological Adaptation

The trabecular architecture of porous tantalum metal in PTTM-enhanced titanium dental implants enhances osseointegration by substantially increasing the three-dimensional bone-implant interfacial area, thereby facilitating angiogenesis and closely replicating native osseous architecture.

Periprosthetic infection remains one of the most challenging clinical issues, often leading to severe outcomes. The introduction of tantalum and its derivatives offers innovative approaches and potential solutions, garnering significant attention in combating periimplantitis. However, studies have shown that tantalum exhibits varying anti-infective effects, both in vitro and in vivo, with its intrinsic antibacterial properties still under debate [26,27]. The biological adaptation of tantalum is characterized by its excellent biocompatibility, ability to support bone growth, resistance to corrosion, and favorable cellular interactions, making it a promising material for a wide range of medical implant applications [26,27].

Tantalum has been demonstrated to interact favorably with various cell types, including osteoblasts, fibroblasts, and endothelial cells. It promotes the adhesion, proliferation, and differentiation of these cells, contributing to the healing process around the implant. This is especially important for tissue regeneration in bone and soft tissue.

Biomechanical Properties

PTTM facilitates elastic deformation and effective load distribution, thereby minimizing localized stress concentration at the bone-implant interface. This load-sharing capability reduces stress transfer and decreases the risk of periprosthetic bone resorption by dispersing mechanical forces throughout the implant structure and the surrounding bone [28].

A summary of the key parameters, observations, and advantages of tantalum dental implants compared to conventional titanium implants is provided in Table 1.

**Table 1:** Advantages of Tantalum Implant Over Conventional Implants

S. No.	Parameter	Observations based on the tantalum implant	Advantage over conventional titanium implant
1	Osseointegration	Bone growth around the tantalum may have enhanced bone integration and promoted its ingrowth into the implant [22,27].	Tantalum implant shows both Osseointegration and Osseoincorporation
2	Adaptability	Tantalum treatment has shown potential for enhancing the biological response to implants, suggesting promising clinical applications [27].	Tantalum-modified implants demonstrated enhanced cell survival of fibroblasts and osteoblasts, reduced soft tissue thickness between the implant and bone, and increased bone formation activity.
3	Bone formation	The porous tantalum midsection of the implant was secured through a fast, intramembranous-like bone healing process. This osseointegration was driven by the development of an osteogenic tissue network, which, over a few months, closely resembles the mean trabecular volumes typically observed in the edentulous posterior jaw [9].	The bone regeneration throughout the porous tantalum trabecular metal network reaches levels comparable to those observed clinically before implant placement, occurring within a few weeks.
4	Mechanical property	The nanomechanical properties of new peri-implant bone adjacent to dental implants with porous tantalum were similar to those of the new bone around implants made entirely of threaded titanium, regardless of the healing time points [10].	The porous tantalum in dental implants may influence the patterns and processes of bone formation around the implant surface.

Uses of tantalum implants

Tantalum’s ability to withstand bodily fluids without corroding, its non-reactivity with human tissues, and its ability to integrate well with bone make it a valuable material in the development of medical implants, surgical instruments, and dental devices. Whether used in orthopedic joint replacements, dental implants, or bone grafting procedures, tantalum offers enhanced performance and longevity, helping to improve the quality of care and outcomes in both medical and dental fields. Some of the uses are listed below.

Implants and Prosthetics

Tantalum is used in dental implants, particularly as a coating on titanium or as part of an alloy. Its ability to integrate with bone tissue helps in the stabilization of implants, promoting better healing and longevity.

It is also used in orthopedic applications, such as joint replacements, including hip and knee replacements. It is highly durable and does not corrode when exposed to bodily fluids, making it ideal for long-term implantation [29].

Surgical Instruments

It is used in the manufacturing of surgical instruments due to its strength, resistance to corrosion, and ability to withstand high temperatures. It is often used for items such as forceps, clamps, and needles [30].

Bone Grafting

Tantalum has been explored for use in bone grafting, particularly in porous granular forms. These forms encourage bone cell growth and help integrate artificial bone with natural bone, making them useful for reconstructive surgery [23,24].

Radiation Shielding

Tantalum is also used in radiotherapy for shielding purposes [31]. It can protect surrounding tissues from radiation during cancer treatments. Tantalum's high resistance to corrosion and excellent biocompatibility make it a suitable material for medical and dental treatments, ensuring the longevity of implants and minimizing complications associated with rejection or corrosion. An investigation examined the protective properties of tantalum (V) oxide-reinforced glass screens on unexposed breast tissue during mammography examinations.

Discussion

Tantalum, an emerging material in dental implantology, has yielded excellent outcomes when compared to conventional titanium implants. Tantalum implants have higher osseointegration and osseoincorporation rates than titanium implants, according to observations. This dual capacity improves integration with the surrounding bone. Al Deeb et al. and Bakri et al. found that tantalum had superior bone ingrowth characteristics, which may contribute to long-term implant durability and success [22,32].

Tantalum's surface characteristics have shown great promise for boosting biological responses. Tantalum-modified implants were linked to higher cell survival rates for osteoblasts and fibroblasts, lower soft tissue thickness, and improved bone formation activity. These properties make tantalum an excellent candidate for therapeutic applications requiring improved tissue contact [32]. In vitro investigations comparing titanium, tantalum, and chromium have demonstrated that tantalum exhibits biocompatibility comparable to that of titanium when assessed using human mesenchymal stem cells. Although titanium has been associated with more rapid cellular proliferation, tantalum appears to preferentially promote osteoblastic differentiation, highlighting its favorable osteogenic profile. However, further research is needed to fully explore its potential, particularly in relation to its antibacterial properties and long-term performance within the body.

Tantalum's porous structure facilitates fast bone growth through intramembranous healing mechanisms. de Arriba et al. discovered that bone repair within the tantalum's trabecular network reaches levels equivalent to pre-implant circumstances in a shorter time. This quick osseointegration demonstrates tantalum's appropriateness for locations that require expedited healing and implant durability [9].

The nanomechanical characteristics of peri-implant bone surrounding tantalum implants are similar to those of titanium implants. Tantalum's porous structure may alter bone development patterns, resulting in improved implant stability. Tantalum's versatility makes it a promising material for a wide range of therapeutic settings, including severe anatomical or physiological circumstances [10]. A study demonstrated that the incorporation of PTTM in knee implants mitigates the resorption of adjacent tibial bone, a common contributor to prosthetic joint failure [33]. The close similarity between the mechanical properties of PTTM and native bone has been shown to limit stress shielding, thereby preserving long-term peri-implant bone integrity. In dental applications, PTTM-enhanced titanium implants retain the established advantages of root-form, endosseous, self-tapping systems, including high primary stability, retrievability, and prosthetic simplicity. When combined with the high biocompatibility, bone-mimetic porous architecture, and favorable mechanical characteristics of PTTM, these implants offer enhanced osseointegration or osseoincorporation, potentially conferring clinical advantages over conventional implant designs.

Tantalum implants provide several benefits over typical titanium implants, including enhanced osseointegration, higher biological adaptability, faster bone regeneration, and comparable mechanical stability. These characteristics suggest that tantalum is a viable material for developing dental implantology. Additional clinical trials and long-term investigations are required to completely confirm its potential and establish standardized guidelines for its application [34].

Limitations of tantalum implants

Tantalum dental implants, while offering advantages such as excellent biocompatibility and strength, also have several limitations. One of the main drawbacks is the high cost of tantalum, making these implants more expensive compared to titanium alternatives. Additionally, there is limited long-term clinical data available regarding the performance of tantalum implants, particularly in the oral environment, which leaves uncertainties about their durability over time. Despite its resistance to corrosion, tantalum may still be prone to degradation under certain conditions in the mouth. The material is also relatively rare and difficult to extract, which can limit its availability and further increase the cost. Although tantalum possesses good mechanical properties, its lower stiffness compared to titanium could lead to stress-shielding effects, potentially impacting bone stimulation and osseointegration. Moreover, while tantalum supports bone growth, the rate of osseointegration may vary, requiring further studies to optimize its use. Fabricating tantalum implants is a more complex process, limiting their widespread adoption in dental practice. Lastly, although rare, there is a potential for allergic reactions or hypersensitivity to tantalum, particularly in patients with sensitivities to metals. These limitations may hinder the broader use of tantalum-based dental implants [35,36].

## Conclusions

Tantalum dental implants represent a promising alternative to traditional materials such as titanium due to their excellent biocompatibility, osteoconductivity, and potential for enhanced bone integration. The unique properties of tantalum, including its porous structure, promote optimal bone growth and provide a favorable environment for osteocyte infiltration, which is crucial for long-term implant success.

However, despite these advantages, several challenges remain. The high cost of tantalum, limited long-term clinical data, potential for material degradation, and fabrication complexities are key factors that may hinder its widespread adoption. Additionally, while tantalum shows good promise in terms of osseointegration, further research is needed to fully understand its behavior in the oral environment, optimize its use, and address any potential limitations, such as stress-shielding effects. Overall, while tantalum dental implants offer significant potential, more studies and advancements in material processing and clinical outcomes are necessary to determine their place in mainstream dental practice.
